# Fine Spatial Scale Variation of Soil Microbial Communities under European Beech and Norway Spruce

**DOI:** 10.3389/fmicb.2016.02067

**Published:** 2016-12-22

**Authors:** Heiko Nacke, Kezia Goldmann, Ingo Schöning, Birgit Pfeiffer, Kristin Kaiser, Genis A. Castillo-Villamizar, Marion Schrumpf, François Buscot, Rolf Daniel, Tesfaye Wubet

**Affiliations:** ^1^Department of Genomic and Applied Microbiology and Göttingen Genomics Laboratory, Institute of Microbiology and Genetics, Georg-August UniversityGöttingen, Germany; ^2^Department of Soil Ecology, UFZ-Helmholtz Centre for Environmental ResearchHalle, Germany; ^3^Department of Biology II, University of LeipzigLeipzig, Germany; ^4^Max Planck Institute for BiogeochemistryJena, Germany; ^5^German Centre for Integrative Biodiversity Research (iDiv) Halle-Jena-LeipzigLeipzig, Germany

**Keywords:** tree species, soil depth, horizontal distance from tree trunk, seasons, soil properties, soil microbial community structure, bacterial 16S rRNA gene, fungal ITS DNA

## Abstract

The complex interactions between trees and soil microbes in forests as well as their inherent seasonal and spatial variations are poorly understood. In this study, we analyzed the effects of major European tree species (*Fagus sylvatica* L. and *Picea abies* (L.) Karst) on soil bacterial and fungal communities. Mineral soil samples were collected from different depths (0–10, 10–20 cm) and at different horizontal distances from beech or spruce trunks (0.5, 1.5, 2.5, 3.5 m) in early summer and autumn. We assessed the composition of soil bacterial and fungal communities based on 16S rRNA gene and ITS DNA sequences. Community composition of bacteria and fungi was most strongly affected by soil pH and tree species. Different ectomycorrhizal fungi (e.g., *Tylospora*) known to establish mutualistic associations with plant roots showed a tree species preference. Moreover, bacterial and fungal community composition showed spatial and seasonal shifts in soil surrounding beech and spruce. The relative abundance of saprotrophic fungi was higher at a depth of 0–10 vs. 10–20 cm depth. This was presumably a result of changes in nutrient availability, as litter input and organic carbon content decreased with soil depth. Overall bacterial community composition showed strong variations under spruce with increasing distance from the tree trunks, which might be attributed in part to higher fine root biomass near spruce trunks. Furthermore, overall bacterial community composition was strongly affected by season under deciduous trees.

## Introduction

Earth currently harbors approximately three trillion trees and only one gram of soil can contain billions of microbial cells (Rosselló-Mora and Amann, 2001; Crowther et al., 2015). The effect of trees on bacteria and fungi in forest soils, comprising many taxa involved in decomposition of plant litter as well as deadwood, is however poorly understood (Wubet et al., 2012; Pfeiffer et al., 2013; Purahong et al., 2014). Forest trees substantially impact soil physical, chemical and biological properties by species-specific stemflow, root architecture, leaf and root litter inputs, root exudates, nutrient uptake, shade, and microclimate (Augusto et al., 2002; Ayres et al., 2009; Raz-Yaseef et al., 2010; Cesarz et al., 2013). As a consequence of direct or indirect tree impacts, changes in the spatial distribution of microbes, vertically through the soil profile as well as horizontally with increasing distance from tree trunks, can occur (Saetre and Bååth, 2000; Ettema and Wardle, 2002). Although numerous studies on the effects of plants on soil microorganisms are available, they rarely focus on microbial communities under trees (Thoms et al., 2010; Urbanová et al., 2015; Uroz et al., 2016). Surveys on effects of pure tree species in a forest stand as well as those focusing on vegetation gradients or chronosequences contributed to the current overall picture concerning tree influences on soil microbial communities (e.g., Cong et al., 2015; Zeng et al., 2016).

European beech (*Fagus sylvatica* L.) and Norway spruce (*Picea abies* (L.) Karst) represent dominant forest trees in Central Europe (Cesarz et al., 2013; Hanewinkel et al., 2013). Since the 19th century, reforestation of devastated forest sites using Norway spruce has been very common in Central Europe (Berger and Berger, 2012). Beech forests show a high seasonal variation in aboveground litter input, which is predominately autumnal. In contrast, the aboveground litter input in spruce forest remains relatively constant over the year. Components of needle litter from Norway spruce such as waxes and phenolic compounds are highly recalcitrant to biological degradation, whereas beech leaf litter contains higher amounts of more readily decomposed water-soluble substances (Nykvist, 1963; Priha and Smolander, 1997). Replacement of beech by spruce species is therefore accompanied by changes in humus form, acidity and soil structure (Berger and Berger, 2012). Upper soil horizons are dominated by leaf litter input, and roots; their residues and exudation patterns shape the subsoil (Moll et al., 2015). Spruce is typically shallow-rooted, whereas beech has a deep rooting system (so called “base-pump”). Consequently, variation in nutrient availability affects microbial communities along soil depths (Huang et al., 2013; Moll et al., 2015). Between *Fagus sylvatica* L. and *Picea abies* (L.) Karst, the quantity and composition of exudates varies with season (Geßler et al., 1998; Fender et al., 2013) and potentially affects microbial processes such as respiration (Cesarz et al., 2013).

European beech and Norway spruce forest stands differ in the magnitude of stemflow. In beech stands, stemflow water contributes 5–20% to the annual soil water input (Koch and Matzner, 1993; Johnson and Lehmann, 2006). Stemflow in conifer forests is often below 1% due to differences in branch angle, specific surface roughness of branches and bark (Johnson and Lehmann, 2006). The high stemflow in beech forests is associated by a decrease of soil pH next to the stem base versus the surrounding soil (Koch and Matzner, 1993). A similar effect has not been demonstrated in Norway spruce forest.

Previous studies have largely used methods providing coarse phylogenetic information to identify effects of forests on soil microbial communities. Using automated ribosomal intergenic spacer analysis (ARISA), ester linked fatty acid methyl ester (EL-FAME) analyses, and denaturing gradient gel electrophoresis (DGGE), differences in soil bacterial and fungal community structure in temperate broad-leaved and coniferous forests have been reported (Lejon et al., 2005; Zechmeister-Boltenstern et al., 2011; Jiang et al., 2012). Recently, Tedersoo et al. (2016) analyzed pyrosequencing-derived ITS sequences to assess the effects of tree diversity on fungi, protists and meiofauna inhabiting forest soil. Results indicated that compared to the effects of individual tree species and soil parameters, tree diversity *per se* had a minor influence on the taxonomic richness of soil biota (Tedersoo et al., 2016). In addition, based on amplicon pyrosequencing data, significant effects of tree species on soil bacterial and fungal community composition were reported by Urbanová et al. (2015).

While several recent marker gene sequencing-based studies focused either on bacteria or fungi in forest soils, they have rarely been considered together (Yarwood et al., 2010; Baldrian et al., 2012; Urbanová et al., 2015). Fungi are typically larger in size than bacteria and exhibit a higher biomass. Therefore, they interact with their environment, e.g., by moving water and nutrients, on a larger spatial scale compared to bacteria (Coleman and Crossley, 1996; van der Heijden et al., 2008; Trevors, 2010), which might result in a more homogeneous distribution of fungal communities in soil. The life cycle of both bacteria and fungi inhabiting forest soils can be strongly affected by seasons through changes in abiotic and biotic factors (Thoms and Gleixner, 2013).

In this study, we applied pyrosequencing of the V3–V5 region of the 16S rRNA gene and the ITS DNA region to assess composition of soil bacterial and fungal communities in a European beech and a Norway spruce forest. We considered potential seasonal variation in microbial communities by collecting samples in early summer and autumn. Furthermore, to determine spatial tree effects, soil collected from different depths and horizontal distances toward tree trunks was considered within this survey. We examined the following hypotheses: (1) bacterial and fungal community composition are affected by tree species, (2) the relative abundance of saprotrophic microorganisms decreases with soil depth, (3) bacteria respond stronger to growing distance from trees than fungi, and (4) seasonal variation of soil bacterial and fungal community composition is stronger under deciduous versus coniferous forests.

## Materials and methods

### Sites and soil sampling

**Figure 1:**
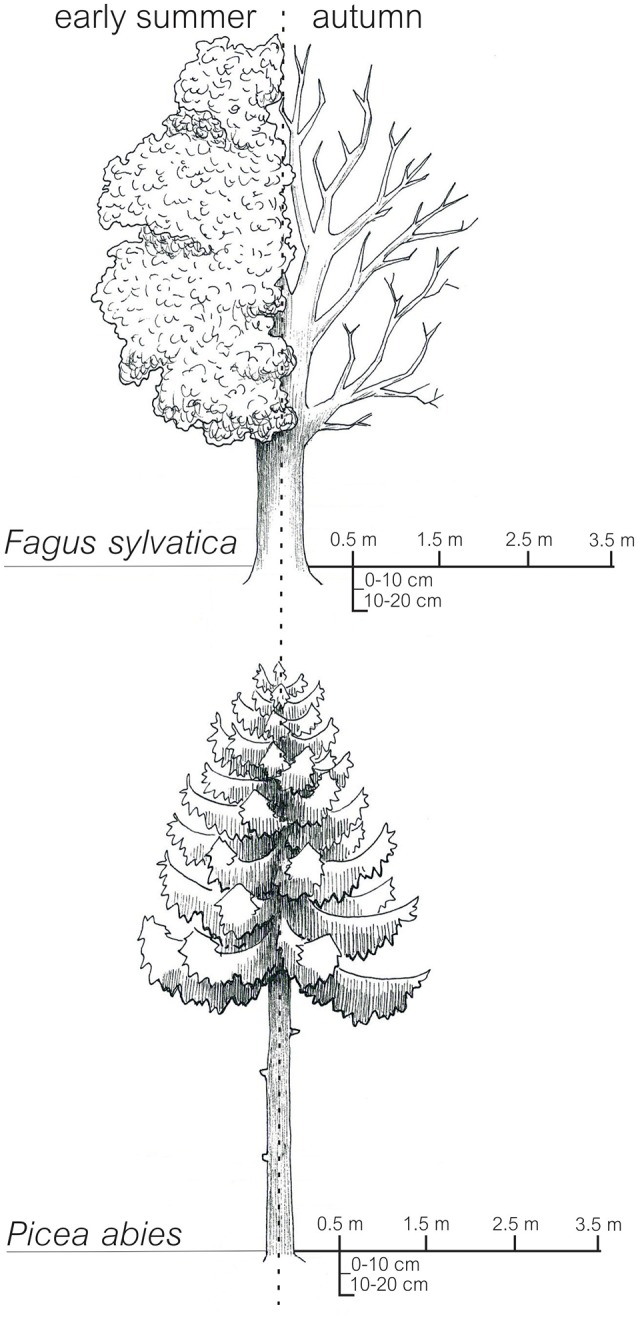
**Sampling design: In early summer and autumn 2012 samples were taken at a distance of 0.5, 1.5, 2.5, and 3.5 m from the tree trunks of four European beech and four Norway spruce trees**. At all sampling points soil samples from 0–10 cm and 10–20 cm were taken.

All soil samples were derived from a beech (*Fagus sylvatica* L.) and a spruce (*Picea abies* L. (Karst)) forest site (distance between the two forest sites: approximately 5 km) located in the Hainich-Dün region in Germany (Fischer et al., 2010). The beech and spruce forest stands were originally established as plantations and are managed (management type, age class forest) since 1760 and approximately 1930, respectively (Wäldchen et al., 2011). Due to the very fertile soils (the original parent material was limestone covered by loess) at both sites, beech-dominated forest would be the natural forest type. The age of the trees at both sites ranged between 50 and 65 y. Beech and spruce trees exhibited average crown radii of 387 ± 29 and 209 ± 12 cm, respectively. The mineral soil was sampled at 0–10 cm and 10–20 cm depth using a split tube sampler with a diameter of 4.8 cm (Eijkelkamp Agrisearch Equipment, Giesbeck, Netherlands). Mineral soil samples were taken from different horizontal distances (0.5, 1.5, 2.5, and 3.5 m) from the trunks of four randomly-selected trees per site (“tree distance”; see Figure 1). Sampling was performed in two seasons, early summer and autumn 2012. Five year averages (2008–2012) of soil temperature, measured at a depth of 10 cm, showed similar seasonal variations in the beech (May: 12.1°C, November: 4.1°C) and spruce forest stand (May: 12.7°C, November: 4.2°C). We applied a paired sampling. The sampling positions in autumn were <30 cm away from the sampling points in early summer (Table S1). All sampling points showed a distance >3.5 m to tree trunks (except trunks of the four selected beech and spruce trees, respectively). In total 128 soil samples (2 sites × 2 seasons × 4 replicate trees × 4 horizontal distances × 2 soil depths) were immediately sieved to <4 mm in the field and individually homogenized. One subsample (>200 g) of each sample was air-dried and sieved to <2 mm for soil chemical analyses and another subsample (50 g) was frozen (−20°C) for extraction of nucleic acids.

### Soil physical and chemical properties

Soil pH was measured in duplicate in the supernatant of 1:2.5 mixtures of soil and aqueous 0.01 M CaCl_2_ with a glass electrode. Additionally, the gravimetric water content of the air-dried soil was determined. The empirical equation of Wäldchen et al. (2012) was used to estimate clay content in the samples. The remaining soil was ground to <100 μm. Ground samples were analyzed for total carbon (TC) and nitrogen (TN) by dry combustion with the CN analyzer “Vario Max”™ (Elementar Analysensysteme GmbH, Hanau, Germany). Inorganic carbon (IC) concentrations were determined with the same analyzer after the ignition of samples for 16 h at 450°C. The organic carbon (OC) concentrations equaled the differences between TC and IC.

### DNA extraction, amplification and pyrosequencing

Total microbial community DNA was extracted from approximately 2 g of frozen soil per sample using the PowerSoil™ total RNA isolation kit, the PowerSoil™ DNA elution accessory kit, and the PowerClean™ DNA Clean-Up kit (MoBio Laboratories, Carlsbad, CA, USA) according to the instruction. DNA concentrations were quantified using a NanoDrop UV-Vis spectrophotometer (Peqlab Biotechnologie GmbH, Erlangen, Germany).

The V3–V5 region of bacterial 16S rRNA genes was amplified by PCR. The following set of primers containing Roche 454 pyrosequencing adaptors (underlined) and a sample-specific MID (Extended Multiplex Identifier) was used: V3for 5′-CCATCTCATCCCTGCGTGTCTCCGACTCAG-MID-TACGGRAGGCAGCAG-3′ (Liu et al., 2007) and V5rev 5′-CCTATCCCCTGTGTGCCTTGGCAGTCTCAGCCGTCAATTCMTTTGAGT-3′ (Wang and Qian, 2009). The PCR reaction mixture (50 μl) contained 10 μl 5-fold reaction buffer (Phusion HF buffer, Thermo Fisher Scientific Inc., Germany), 200 μM of each of the four deoxynucleoside triphosphates, 5% DMSO, 1 U Phusion high fidelity DNA polymerase (Thermo Fisher Scientific Inc.), approximately 25 ng DNA as template, and 4 μM of each of the primers. The PCR reactions were initiated at 98°C (2 min), followed by 25 cycles of 98°C (45 s), 58°C (45 s), and 72°C (40 s), and ended with incubation at 72°C for 5 min.

Fungal ITS DNA was amplified using primer ITS1F (Gardes and Bruns, 1993) containing a sample-specific MID and Roche 454 pyrosequencing adaptor B and primer ITS4 (White et al., 1990) containing Roche 454 pyrosequencing adaptor A. The PCR reactions were performed in a total volume of 50 μl reaction mix containing 1 μl DNA template (7–15 ng), 25 μl Go Taq Green Master mix (Promega, Mannheim, Germany) and 1 μl 25 pmol of each of the ITS region-specific primers. Touchdown PCR conditions as described by Wubet et al. (2012) were used to amplify fungal ITS DNA.

All samples were amplified in triplicate, purified using the peqGold gel extraction kit (Peqlab Biotechnologie GmbH) and the Qiagen gel extraction kit (Qiagen, Hilden, Germany) as recommended by the manufacturer, and pooled in equal amounts. Quantification of PCR products was performed using the Quant-iT dsDNA BR assay kit and a Qubit fluorometer (Life Technologies GmbH, Karlsruhe, Germany). Sequences of partial 16S rRNA genes and fungal ITS DNA were decoded at the Göttingen Genomics Laboratory and the Department of Soil Ecology (UFZ-Helmholtz Centre for Environmental Research, Halle, Germany), respectively, using a Roche GS-FLX 454 pyrosequencer (Roche, Mannheim, Germany) and Titanium chemistry as recommended by the manufacturer.

The 16S rRNA gene and ITS DNA sequences were deposited in the National Center for Biotechnology Information (NCBI) Sequence Read Archive (SRA) under study accession numbers SRP040766 and SRP044665, respectively.

### Sequence analysis

Bacterial 16S rRNA gene sequence datasets were preprocessed as described by Broszat et al. (2014). Briefly, bacterial sequences shorter than 200 bp, as well as those exhibiting low quality values (<25), more than two primer mismatches, or long homopolymers (>8 bp), were removed using QIIME (Caporaso et al., 2010). In addition, the bioinformatics tools cutadapt (Martin, 2011), Uchime (Edgar et al., 2011), and Acacia (Bragg et al., 2012) were used for truncation of remaining primer sequences, removal of potential chimeric sequences, and removal of noise introduced by amplicon pyrosequencing. Uclust (Edgar, 2010), implemented in QIIME (Caporaso et al., 2010), was used to determine bacterial OTUs at a genetic distance of 3%. To taxonomically classify OTUs, partial 16S rRNA gene sequences were compared with the SILVA SSU database release 119 (Pruesse et al., 2007). OTUs classified as chloroplast or mitochondrion and unclassified OTUs (proportion of unclassified OTUs was approximately 0.2%), which were not affiliated to bacteria, were removed from 16S rRNA gene sequence datasets.

Fungal ITS DNA sequence datasets were preprocessed with Mothur (Schloss et al., 2009) as described by Goldmann et al. (2015). In brief, sequences with ambiguous bases, homopolymers and primer differences (>8 bp) as well as MIDs were removed in a first filtering step. Simultanously, short reads (<300 bp), sequences with a low quality score (<20) and noisy sequence ends were removed. Samples were checked for chimeric sequences using the UCHIME algorithm (Edgar et al., 2011). Cd-hit (Li and Godzik, 2006) was applied to determine fungal OTUs at 3% genetic distance. To identify fungi and taxonomically classify OTUs, ITS DNA sequences were queried against the UNITE database (Kõljalg et al., 2013) by using the classify.seq command as implemented in MOTHUR (Schloss et al., 2009). All produced OTUs belonged to the kingdom fungi. To improve the taxonomical resolution, OTUs that had been assigned only down to the family level were subjected to a BLASTn search (e.g., Johnson et al., 2008) against the NCBI GenBank database (Benson et al., 2015). The searches excluded uncultured and environmental sample sequences and only assignments with a query cover >95%, *E* <0.0001 and sequence identity >97% were considered. Finally, all fungal OTUs identified at the genus level were grouped into ectomycorrhizal, saprotrophic, and other fungi based on literature.

Bacterial and fungal OTUs comprising only one or two sequences (singleton and doubleton OTUs) were removed from the datasets. The number of analyzed sequences per sample can have an effect on the predicted number of OTUs (Morales et al., 2009). Therefore, OTU-based comparisons were performed at the same level of surveying effort (bacteria: 2540 sequences per sample; fungi: 1996 sequences per sample). In this study, we focused on microbial community composition. Data on microbial diversity is provided in the Supplementary Material (see Figures S1, S2). OTUs identified at a genetic distance of 3% were used to calculate rarefaction curves and the Shannon index.

### Statistical analyses

The response of main soil characteristics (e.g., C:N ratio, clay content) to soil depth (0–10 and 10–20 cm depth), season (early summer and autumn) and tree distance (0.5, 1.5, 2.5, and 3.5 m) was assessed for both study sites separately by analysis of covariance (ANCOVA) using the “aov” command of the “Stats” R-package (R Development Core Team, 2015). The random effects of the four sampling transects per study site were considered in the analysis by including them as a factor in our linear models (tree replicate).

The effect of tree species on soil bacterial and fungal community composition, respectively, was visualized using principal coordinates analysis plots generated with the emperor software package (Vázquez-Baeza et al., 2013) and the “ordiplot” function incorporating environmental vectors calculated with the “envfit” function of the “Vegan” R-package (Oksanen et al., 2016). In order to test the effects of tree replicate, soil pH, OC, soil depth, sampling season, and tree distance on bacterial and fungal community composition, we performed multivariate analysis of variance (MANOVA) using the “adonis" command of the “Vegan” R-package (Oksanen et al., 2016) based on weighted UniFrac (Lozupone et al., 2011) distance matrices. The adonis function in R implements a sequential sum of squares (type 1). A priori we decided to include first the random variance of the tree replicates and important abiotic drivers (soil pH and organic C) into the model. In a second step the factors depth, season and distance were added. This means that the significance of depth, season and distance was examined after removal of variance explained by soil pH and organic C concentration. Changing the order of soil pH and organic C or the order of depth, season and distance in the model would not change the significance of the individual factors. This can be explained by the missing collinearity among these factors. These analyses were conducted for whole microbial communities and microbial communities under each tree species individually. Adjusted *R*^2^-values of total models increased with the addition of every single considered parameter (Tables S2, S3).

To further identify individual taxa strongly associated with a specific tree species, season or spatial position in soil, the multipatt algorithm and the “IndVal” function in the “Indicspecies” R-package (De Cáceres and Legendre, 2009) was used based on bacterial and fungal OTUs. The PAST statistical package (Hammer et al., 2001) was used for the performance of Mann-Whitney U test and Spearman's rank correlations. We applied Mann-Whitney U test to identify dominant genera showing significant differences between sets of samples. Spearman's rank correlations were used to correlate relative abundances of dominant genera with soil parameters.

## Results

### General characteristics of soil samples

Both forest stands grow on limestone, which is covered with a loess layer of variable thickness. The loess layer is thinner at the spruce than at the beech forest site. Therefore, in 0–10 cm depth pH values ranged between 3.1 and 5.9 at the spruce site and between 3.7 and 4.4 at the beech site (Table 1, Table S4). The pH values determined for our samples are typical for the two forest sites. At 5 out of 32 sampling locations within the spruce forest the pH at a depth of 0–10 cm was >5.5 indicating that the loess layer was less pronounced or absent and that the parent material mainly originated from limestone. We did not detect a decrease of the soil pH next to the stem basis of beech trees (0.5 m tree distance) compared to the other considered sampling distances (Table S4). At a depth of 10–20 cm the average pH increased by 0.9 units in the spruce stand, whereas it decreased by 0.2 units in the beech stand, which is again a result of the lower loess layer thickness in the spruce compared to the beech stand. This was confirmed by the clay content (0–10 cm), which was with 388 ± 15.2 g kg^−1^ (mean ± standard error) on average higher at the spruce than at the beech site with 276 ± 4.4 g kg^−1^. At the 0–10 cm depth, the soils contained on average 32.6 ± 2.3 g kg^−1^ and 26.2 ± 0.8 g kg^−1^ OC in the spruce and beech stand. The OC concentrations decreased with depth. Organic C concentrations at the 0- to 10-cm depth were strongly related to estimated clay contents (*r* = 0.79, *P* < 0.001). Due to collinearity between OC concentration, clay content, and C:N ratio, we only included OC concentration in subsequent statistical analyses.

**Table 1:** **Basic properties of soil samples derived from the beech and spruce stands**.

**Origin**	**Soil depth**	**pH**	**Clay content [g kg ^−1^]**	**Organic C [g kg ^−1^]**	**C:N ratio**
Beech stand	0–10 cm	4.0 ± 0.0	276 ± 4.4	26.2 ± 0.77	12.0 ± 0.10
Beech stand	10–20 cm	3.8 ± 0.0	249 ± 4.2	14.5 ± 0.55	11.0 ± 0.11
Spruce stand	0–10 cm	4.0 ± 0.2	388 ± 15.2	32.6 ± 2.30	14.8 ± 0.27
Spruce stand	10–20 cm	4.9 ± 0.2	380 ± 14.0	15.1 ± 0.65	11.0 ± 0.20

*Mean values and standard errors are provided for pH, clay content, organic C, and C:N ratio*.

### Soil bacterial and fungal community profiles

**Figure 2:**
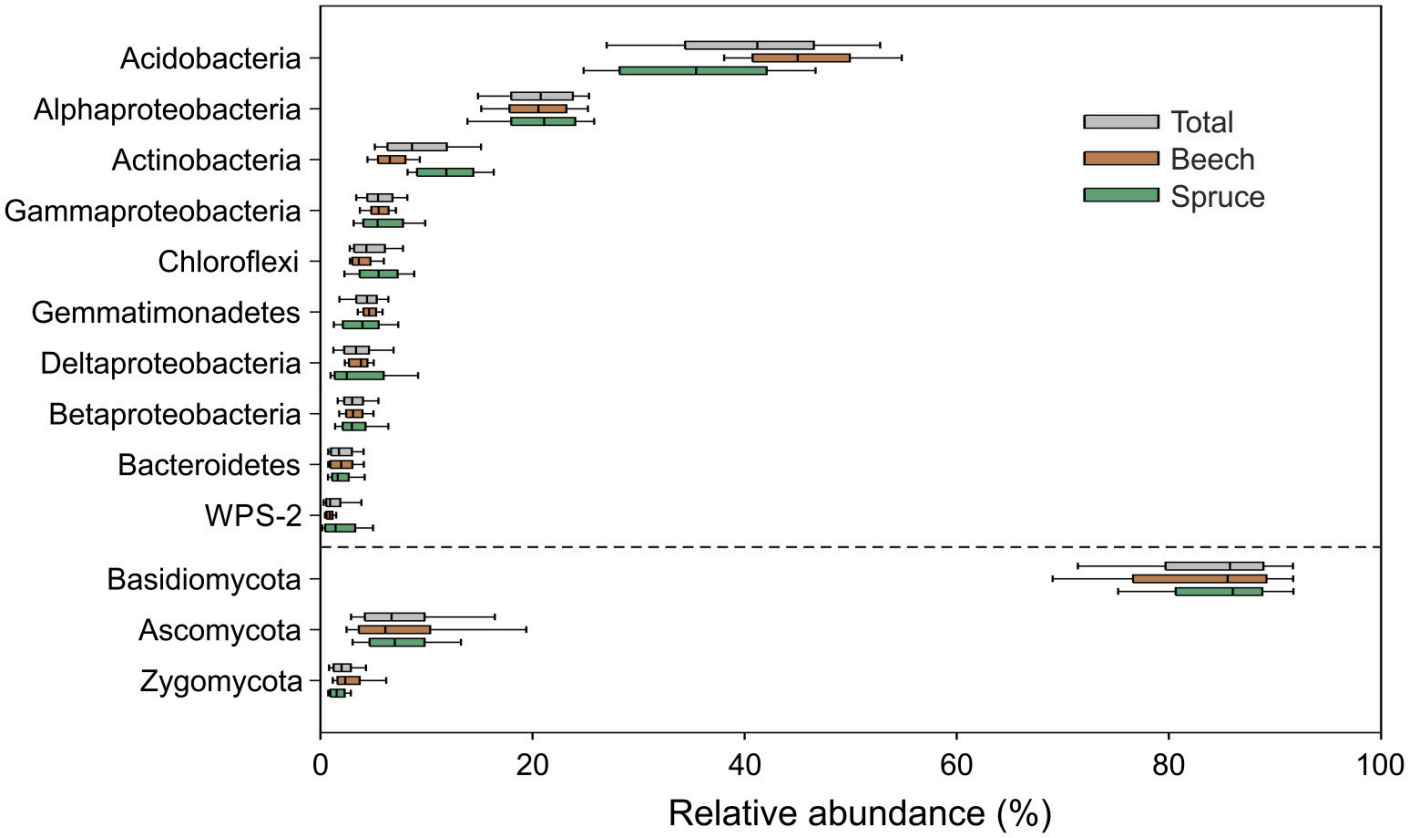
**Box-and-whiskers plot showing relative abundances of bacterial and fungal phyla as well as proteobacterial classes detected in each of the analyzed 128 soil samples**. Relative abundances of taxa across all samples (gray color) as well as separately with respect to soil surrounding beech (brown color) and spruce (green color) are depicted. The dashed line separates relative abundances of bacterial and fungal taxa.

**Figure 3:**
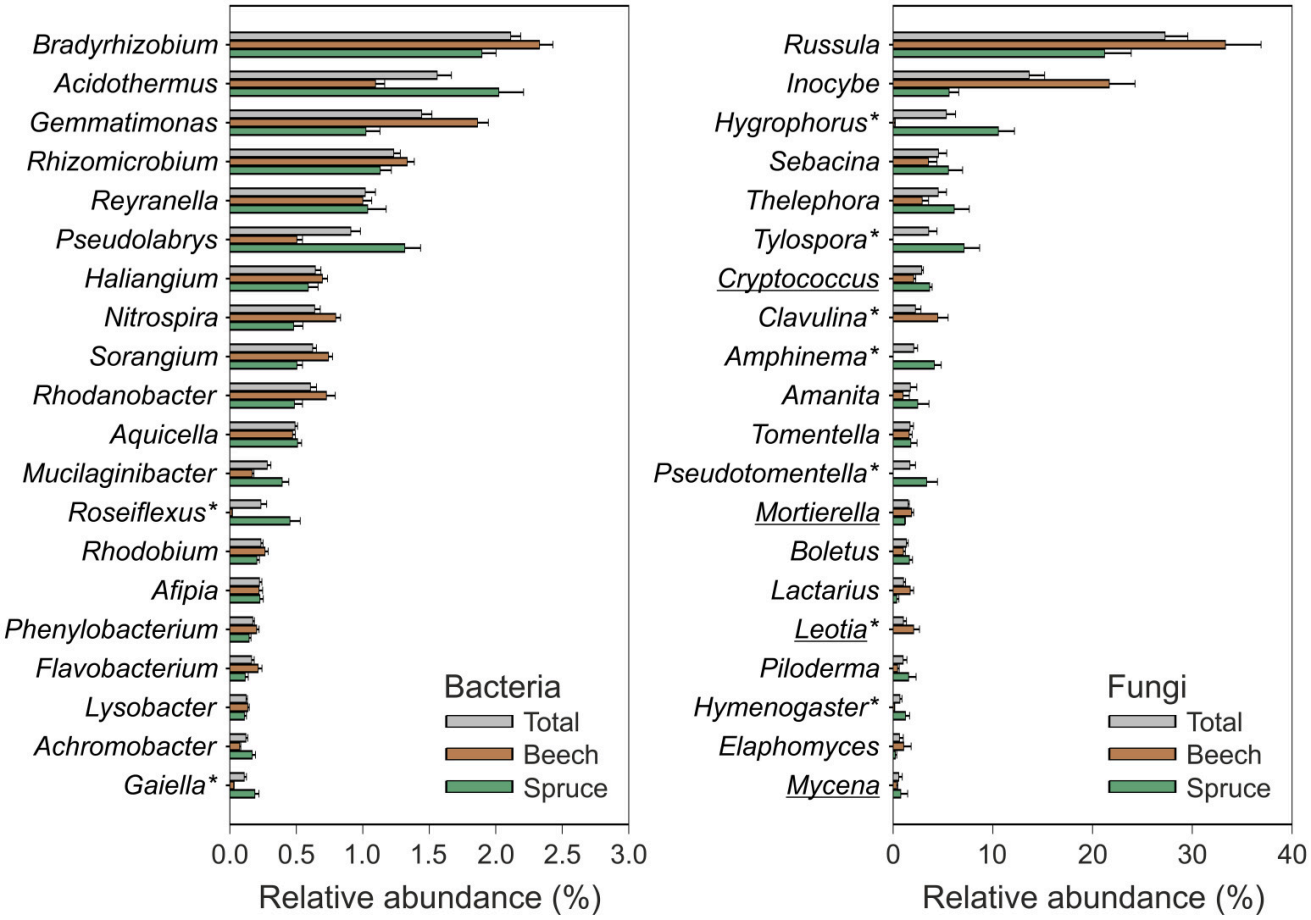
**Relative abundance of dominant bacterial and fungal genera detected in the analyzed soil samples**. The data represent mean values and standard errors of relative abundance for the 20 most abundant bacterial and fungal genera, respectively. Acidobacteria were analyzed at the subgroup level and therefore not considered within this figure. Relative abundances of taxa across all samples (gray color) as well as separately with respect to soil surrounding beech (brown color) and spruce (green color) are depicted. Asterisks indicate taxa showing an at least five-fold difference in mean relative abundance between spruce and beech (*P* < 0.001 for the Mann-Whitney U test). Underlined taxa: saprotrophic fungi (all other depicted fungal genera represent ectomycorrhizal fungi).

Pyrotag processing yielded a total of 864,096 bacterial and 255,488 fungal high-quality sequences with an average length of 464 and 300 bp, respectively. At a genetic distance of 3%, 23,727 bacterial and 1336 fungal OTUs were identified across all analyzed soil samples. In the final microbial dataset, the number of OTUs per individual soil sample ranged from 505 to 1440 (bacteria) and 45 to 191 (fungi). Taxonomic classification was based on closest matches of OTUs to particular phylogenetic groups. Each of the dominant phyla and genera identified in this study (see Figures 2, 3) is represented by more than one OTU determined at a genetic distance of 3%. The bacterial phyla and proteobacterial classes detected in each of the individual soil samples comprised Acidobacteria (average relative abundance: 40.7 ± 0.8%), Alphaproteobacteria (20.5 ± 0.4%), Actinobacteria (9.4 ± 0.3%), Gammaproteobacteria (5.8 ± 0.2%), Chloroflexi, (4.8 ± 0.2%), Gemmatimonadetes (4.4 ± 0.2%), Deltaproteobacteria (3.8 ± 0.2%), Betaproteobacteria (3.3 ± 0.1%), Bacteroidetes (2.1 ± 0.1%) and candidate division WPS-2 (1.5 ± 0.1%) (Figure 2). Genus level analysis of the bacterial community showed high relative abundances (average relative abundance of each genus >1%) of *Bradyrhizobium* followed by *Acidothermus, Gemmatimonas, Rhizomicrobium*, and *Reyranella* (Figure 3). Acidobacteria represent the most abundant phylum in our study. Subgroup 2 (average relative abundance: 14.1 ± 0.6%), subgroup 1 (11.1 ± 0.5%), subgroup 3 (10.1 ± 0.3%), and subgroup 6 (2.8% ± 0.3%) showed the highest average relative abundance among acidobacterial representatives.

The fungal community was dominated by Basidiomycota (average relative abundance: 87.7 ± 0.7%), followed by Ascomycota (8.9 ± 0.6%), and Zygomycota (2.5 ± 0.2%) (Figure 2). In total, 89% of all dominant fungal OTUs were assigned to more than 200 fungal genera. The most abundant fungal genera were *Russula* (average relative abundance: 33.3 ± 2.7%), followed by *Inocybe* (16.8 ± 1.8%), *Hygrophorus* (6.2 ± 1.0%), *Sebacina* (5.7 ± 1.0%), and *Thelephora* (5.6 ± 1.0%) (Figure 3). Functional group assignment of the fungal communities revealed that among the 20 most abundant fungal genera (Figure 3), 16 are known to be ectomycorrhizal (ECM) fungi, whereas the remaining four have a saprotrophic lifestyle (*Cryptococcus, Mortierella, Leotia*, and *Mycena*).

### Tree species effects on microbial community composition

**Figure 4:**
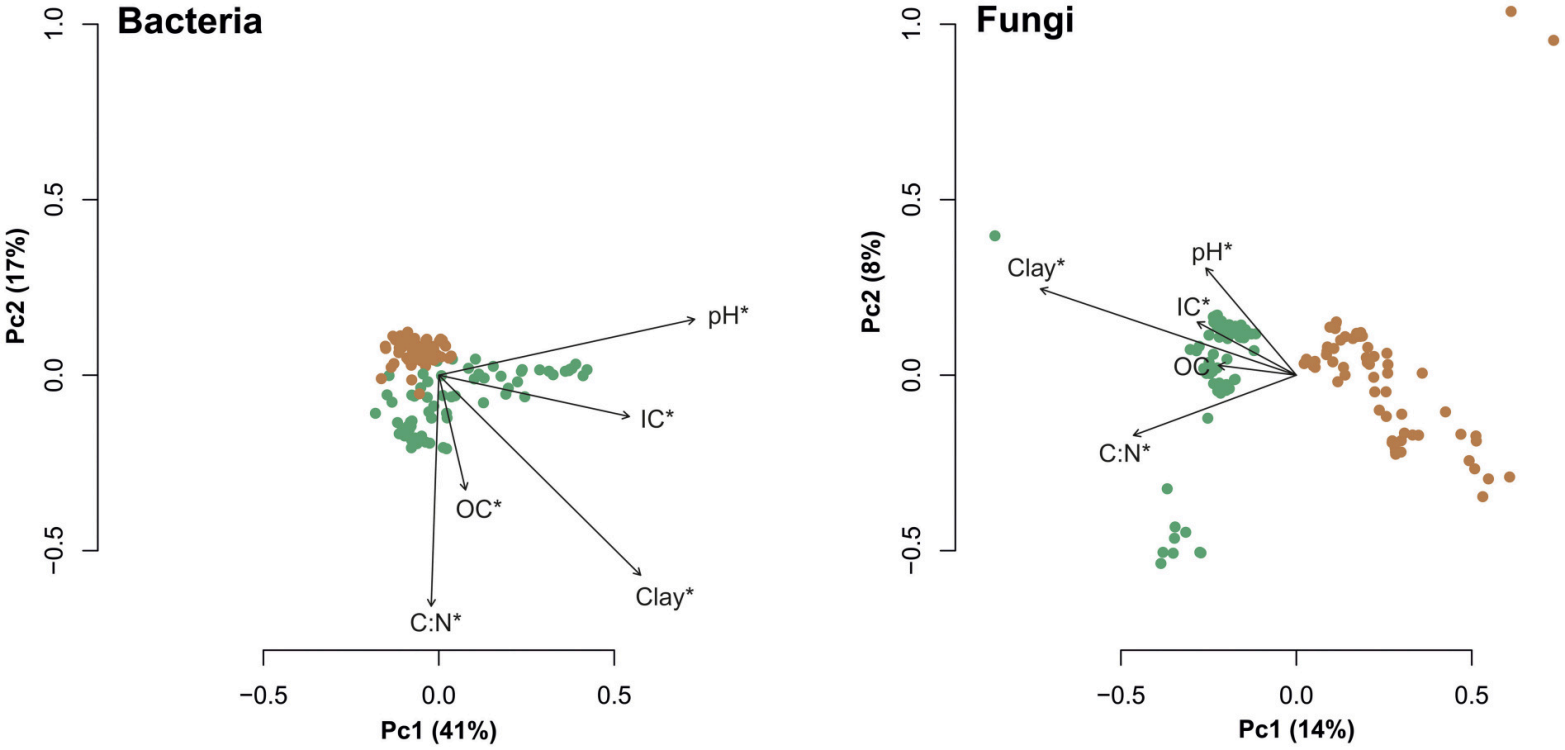
**Principal coordinates analysis plots based on weighted UniFrac distances calculated at 3% genetic distance**. Brown circles represent samples derived from beech surrounding soil and samples derived from spruce surrounding soil are depicted as green circles. Vectors represent response variables pH, estimated clay content, C:N ratio, organic carbon (OC), and inorganic carbon (IC). Significant values (*P* < 0.05) according to “envfit” calculations are indicated by asterisks.

Samples collected under beech and spruce tend to cluster separately in principal coordinates analysis plots (Figure 4). The axes of these plots explain less of the variability in fungal community composition (axis 1 = 14%) compared to bacterial community composition (axis 1 = 41%). The variation explained by tree species was 13.8% (*P* < 0.001) in bacterial and 14.9% (*P* < 0.001) in fungal communities (Table S2). Furthermore, tree species (European beech or Norway spruce) had a stronger impact on soil bacterial and fungal community composition than soil depth, distance from tree trunk or season (Table S2). We identified specific indicator OTUs for soils surrounding beech or spruce stands (Table S5). Each bacterial indicator OTU showed an average relative abundance <1%, whereas few fungal indicator OTUs showed relative abundances >1%. Detailed information on relative abundances for all indicator OTUs is provided in Table S5.

For bacteria, 13 indicator OTUs were determined at the beech site and 10 indicator OTUs at the spruce site. The majority of bacterial OTUs representing indicators at the beech site were affiliated to Acidobacteria (mainly subgroup 2) (Table S5). Indicators at the spruce site comprised Chloroflexi, WD272 and several Acidobacteria subgroup 1 OTUs.

For both tree species, eight fungal OTUs were identified as potential indicators (Table S5). Under beech, a saprotrophic *Mortierella elongata* OTU and a *Trichoderma* OTU and ECM fungi OTUs (a *Russula cyanoxantha* OTU and a *Xerocomus chrysenteron* OTU) were identified as indicator OTUs. Indicators for spruce were three OTUs classified as saprotrophic fungi (*Exophiala* and two *Penicillium* OTUs). The two indicator ECM fungi under spruce were *Hygrophorus* and *Amphinema*.

Microbial community composition under both tree species was significantly affected by tree replicate, soil pH and OC (Table 2). Among the analyzed factors soil pH and tree species explained most of the variation in microbial community composition (Table S2).

**Table 2:** **Multivariate analysis of variance based on weighted UniFrac distances with tree replicate, pH, OC, soil depth, season and distance as response variable**.

	***df***	**Beech stand**				**Spruce stand**			
		**Bacterial community**		**Fungal community**		**Bacterial community**		**Fungal community**	
		***MS***	***R*^2^**	***MS***	***R*^2^**	***MS***	***R*^2^**	***MS***	***R*^2^**
Tree replicate	3	0.027	0.063^**^	1.637	0.233^***^	0.201	0.171^***^	2.227	0.323^***^
pH	1	0.221	0.171^***^	0.679	0.032^**^	0.385	0.109^***^	0.987	0.048^***^
OC	1	0.124	0.097^***^	0.843	0.04^***^	0.069	0.020	0.485	0.023^**^
Depth	1	0.016	0.013	0.277	0.013	0.100	0.028^*^	0.260	0.013
Season	1	0.078	0.06^***^	0.445	0.021	0.077	0.022	0.422	0.02^*^
Distance	1	0.026	0.021^*^	0.427	0.020	0.261	0.074^***^	0.672	0.032^***^
Residuals	55	0.014	0.576	0.245	0.640	0.037	0.575	0.204	0.541

Explanatory variables are given in rows in the order of entering the analysis. This table presents degrees of freedom (df), mean squares (MS), and R^2^-values. Significant results are indicated by

* *P <0.05*,

** *P <0.01*,

*** *P <0.001*.

### Spatial and seasonal variability of soil microbial community composition

**Figure 5:**
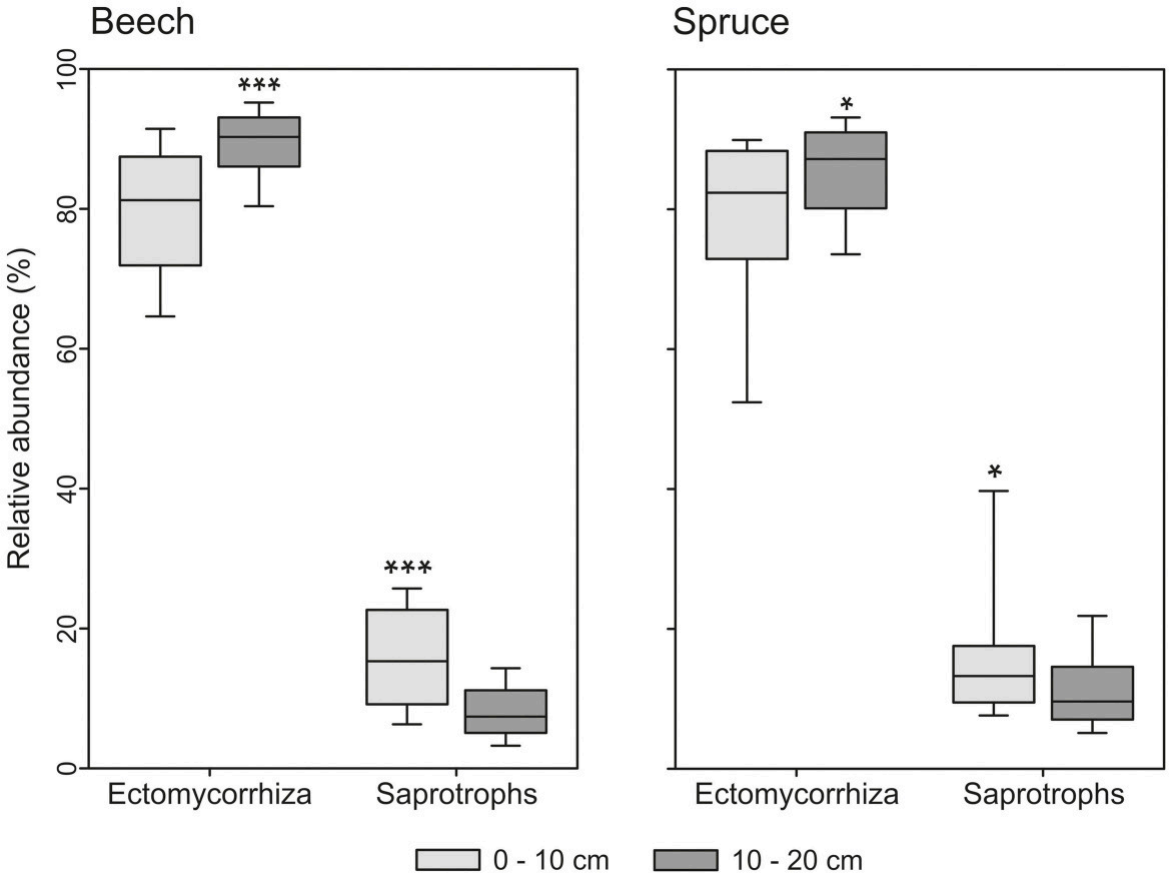
**Box-and-whiskers plots showing relative abundance of ectomycorrhizal and saprotrophic fungi under beech and spruce in relation to soil depths**. The asterisks indicate significant differences between soil depths for each ecological group determined by ANOVA; ^*^significant (*P* < 0.05), ^***^highly significant (*P* < 0.001).

Bacterial community composition varied significantly with depth under spruce (Table 2). We found that relative abundance of OTUs of the dominant genus *Gaiella* was negatively correlated with OC concentration (*P* < 0.001) and higher at the 10- to 20-cm depth than the 0- to 10-cm depth. The relative abundance of the bacterial genus *Mucilaginibacter* also showed variations with soil depth. It was higher at the 0- to 10-cm depth vs. the 10- to 20-cm depth (*P* < 0.001). The fungal community composition showed no correlation with soil depth under both tree species (Table 2). However, the detected saprotrophic fungi were associated with the upper (0–10 cm depth) mineral soil layers, which were rich in OC (Figure 5). Additionally, the indicator species analysis identified mainly saprotrophic OTUs in the upper 10 cm of the studied soils (Table S5).

Spatial horizontal variation of overall bacterial community composition was significant in soil under beech (*P* < 0.05) and under spruce (*P* < 0.001) (Table 2). We found that relative abundance of the dominant bacterial genus *Nitrospira* was significantly higher at 3.5 m vs. 0.5 m distance from spruce trees. Furthermore, a Nitrospirales OTU was identified as an indicator for tree distances of 2.5 and 3.5 m (Table S5). Under beech trees, the relative abundance of *Pseudolabrys* differed significantly between 0.5 m and 3.5 m horizontal tree distance. Higher relative abundance was detected in soil located close to tree trunks. This effect was recorded with respect to both analyzed soil depths (*P* < 0.05). Overall fungal community composition differed significantly at different horizontal tree distances only in soil of the spruce stand (*P* < 0.01) (Table 2). However, fungal indicator species for certain combinations of tree distances were found in beech (Table S5) and spruce stands (Table S5).

A significant seasonal effect on bacterial community composition was detected in soil under beech (*P* < 0.001) (Table 2). Sequences corresponding to the Rhizobiales (*Bradyrhizobium* and *Rhodobium*) showed significantly higher relative abundance in autumn versus early summer (*P* < 0.001). Consistently, the analysis of indicator species identified an OTU affiliated to *Bradyrhizobium* in soil under beech in autumn (Table S5). A seasonal impact on fungal community composition was found in soil of the spruce stand (*P* < 0.05) (Table 2). Two fungal indicator species were identified in early summer in the spruce stand (Table S5). Fungal indicator species for both seasons (autumn and early summer) occurred under beech (Table S5).

## Discussion

### Selective association of tree species, bacteria, and fungi

Differences in distribution of microbial taxa were identified between soil under beech and spruce. This was expected, as even tree genotype within a species can have significant impacts on microbial communities (Schweitzer et al., 2008). A Chloroflexi OTU was identified as indicator for soil surrounding spruce. As several potential genes involved in phytochemical breakdown have been identified in Chloroflexi (Hug et al., 2013; Houghton et al., 2015), it is possible that this indicator microorganism plays a role in decomposition of spruce litter. Furthermore, the occurrence of several members of Acidobacteria was significantly affected by tree species. It can be assumed that acidobacterial taxa contribute to decomposition in forest soils, as genomic and culture characteristics of subgroup 1 and 3 strains have been shown to utilize plant-derived biopolymers (Ward et al., 2009; García-Fraile et al., 2015). Shifts in occurrence of acidobacterial representatives between soil under European beech and Norway spruce might imply preferences for leaf or needle litter. A study on composition of bacterial communities under different deciduous and coniferous trees (e.g., *Picea* and Fagales species) in Czech forest stands also indicated litter preferences of Acidobacteria (Urbanová et al., 2015).

Forest vegetation (in particular dominant tree species) is important for distribution of mutualistic and saprotrophic fungi (Lauber et al., 2008; Goldmann et al., 2015). ECM fungi (e.g., *Russula, Inocybe, Piloderma*) establish mutualistic associations with plant roots (Smith and Read, 2008) and show preferences for particular tree species (Ishida et al., 2007; Thoms et al., 2010). In accordance with our study, Goldmann et al. (2015) and Miyamoto et al. (2015) reported a preference of *Tylospora* for coniferous trees. Some identified fungal indicators under beech (*Mortierella elongata, Trichoderma, Russula cyanoxantha*) are known to be widespread not just under a certain tree species (Wuczkowski et al., 2003; Grebenc and Kraigher, 2007; Nagy et al., 2011). In contrast, the ECM fungus *Xerocomus chrysenteron* is known to have a preference for beech (Shi et al., 2002). Indicator species for spruce included three OTUs classified as saprotrophic fungi. *Exophiala* has already been described as a fungal genus decaying leafs in rainforests (Polishook et al., 1996) or existing as rhizospheric associates in temperate sites (Summerbell, 2005). Another two *Penicillium* OTUs were identified as saprotrophic indicators for spruce. Previous research (Johansson and Marklund, 1980) reported *Penicillium* to be antagonistic to *Fomes*, a well-known fungus infecting spruce trees (Schmidt, 2013). The indicative ECM fungi under spruce, *Hygrophorus* and *Amphinema*, were abundant and previously described for spruce ecosystems (Scattolin et al., 2008; Velmala et al., 2013).

Under both tree species, microbial community composition was significantly affected by pH and OC concentration. Noteworthy, among the analyzed factors soil pH and tree species explained most of the variation in overall community composition of bacteria and fungi. Several previous studies have identified soil pH as a major driver of soil bacterial community composition across different regions and land use types (e.g., Lauber et al., 2009; Nacke et al., 2011). In accordance with our results, pH also explained a substantial fraction of variance in microbial community composition within other deciduous and coniferous forest soils (Lauber et al., 2009; Thoms et al., 2010; Goldmann et al., 2015). Furthermore, experiments including addition of substrates such as cellulose, lignin, and glucose to soil showed that the quantity of OC can have a significant impact on soil microbial community composition (Nakatsu et al., 2005; Goldfarb et al., 2011).

### Relative abundance of saprotrophic fungi decreases with soil depth

Previous surveys based on DGGE analysis as well as Sanger sequencing and pyrosequencing of 16S rRNA genes have revealed differences in bacterial community composition between topsoil and subsoil (Hansel et al., 2008; Eilers et al., 2012; Huang et al., 2013). This is a result of changes in soil characteristics such as organic C or N concentrations along soil profiles (Hansel et al., 2008; Will et al., 2010). Consistently, relative abundances of the bacterial genus *Gaiella*, which were higher in 10–20 cm depth than in 0–10 cm depth, were negatively correlated with organic C concentration. Different *Mucilaginibacter* representatives are capable of pectin, xylan, and laminarin degradation (Pankratov et al., 2007). *Mucilaginibacter* was more abundant in topsoils (0–10 cm). The genus has been previously associated with cellulose decomposition based on stable isotope probing (Štursová et al., 2012). Leaf and needle litter contains high amounts of the plant cell wall components xylan, pectin, and cellulose, and enters the upper mineral soil first, perhaps explaining the distribution of *Mucilaginibacter* OTUs.

Recently, McGuire et al. (2013) found discrete fungal communities in different soil horizons in boreal and tropical forest. This can be explained by changing carbon and nutrient contents in soil combined with fungal enzymatic decay abilities (McGuire et al., 2010; Prescott, 2010). Our results (Table 2) showed that fungal taxa in temperate forests do not underlay similar mechanisms as found previously. However, we identified different saprotrophic fungi showing preferences for the upper (0–10 cm depth) mineral soil layer, which was rich in OC. Influenced by the litter layer, the upper 10 cm show high habitat heterogeneity, competition amongst fungi for space, carbon and other soil nutrients (Kadowaki et al., 2014). ECM fungal taxa receive carbon through mycelium connected to plant roots (Smith and Read, 2008). In this study, ECM fungi were abundant irrespective of soil depth since these fungi are not C-limited and may colonize deeper soil layers (McGuire et al., 2013).

### Bacteria are affected by horizontal tree distance under beech and spruce

Soil microbial community composition showed higher variability with respect to tree distance under spruce trees versus beech. It is known that spatial distribution of soil microbes can reflect the zone of influence and positioning of individual trees in forests (Saetre and Bååth, 2000; Ettema and Wardle, 2002). As stemflow was shown to significantly decrease soil pH, specifically close to beech trees (Koch and Matzner, 1993), we expected a clear change in microbial community composition next to beech trunks (0.5 m tree distance). However, we could neither detect a decrease in pH at 0.5 m distance to beech trunks, nor a strong change in microbial community composition next to the beech trees. Spatial horizontal variations in bacterial community composition under beech and spruce, recorded in this study, might have been partly evoked by changes in root activities with respect to varying tree distances. N demand of spruce trees in summer and autumn is mainly met by uptake of N compounds from soil and subsequent transport of reduced N from the roots to the shoot via the transpiration stream (Weber et al., 1998). Due to a negative relationship between fine root biomass and tree distance (steep decrease of fine root biomass at tree distances >2 m) (Petritan et al., 2011), uptake of N compounds via roots might be more pronounced in soil located close to the analyzed coniferous tree trunks. This potentially explains the spatial horizontal variations in occurrence of nitrifying bacteria belonging to Nitrospirales under spruce.

Under beech, relative abundance of *Pseudolabrys* was significantly affected by horizontal tree distance. Only one *Pseudolabrys* species, isolated from Taiwanese soil, has been described (Kämpfer et al., 2006). In our study, more than one OTU determined at a genetic distance of 3% was affiliated to *Pseudolabrys*. The taxon *Pseudolabrys*, representing one of the most abundant genera detected in this study, belongs to the Rhizobiales, which are known to interact with plants (Erlacher et al., 2015). Changes in root densities or activities may be a major reason for high relative abundance of *Pseudolabrys* in soil located close to beech trunks.

Branco et al. (2013) found that an increase in soil pH with pine tree distance was related to changing occurrence of fungal species. Variation in pH at different tree distances (Table S4) also account for changes in fungal community composition under the conifer trees analyzed in our study (*P* < 0.05) (Table 2).

### More seasonal soil community variation in beech than in spruce forests

Soil bacterial community composition under beech was strongly affected by season (*P* < 0.001). Recently, López-Mondéjar et al. (2015) reported that bacterial communities undergo seasonal changes in mineral soil of a *Quercus petraea* (Matt.) Liebl forest. They assume that seasonal differences in the activity of tree roots are a major driver of soil bacterial community composition in deciduous forest. Here, we found that different members of the Rhizobiales were more abundant under beech in autumn than in early summer. As Rhizobiales are known to interact with plants, seasonal root impacts might affect their abundance in temperate deciduous forest. Understory vegetation varies between European beech and Norway spruce age class forests in the study region (Boch et al., 2013). It is possible that the Rhizobiales community is affected by seasonal changes in understory vegetation. Furthermore, seasonal shifts in soil moisture and temperature may also affect bacterial community composition in the analyzed soil (Kaiser et al., 2010; Shay et al., 2015).

Seasonal impacts on fungi were reported previously (e.g., Stevenson et al., 2014; Moll et al., 2015). In this study, soil fungal community composition was affected by season under spruce (*P* < 0.05) but not as expected under beech. Recently, Voříšková et al. (2014) also detected no significant seasonal effect on fungal community composition in soil of a deciduous forest (oak forest near Prague, Czech Republic). Nevertheless, in the litter horizon, which was not analyzed in our study, seasonal changes in fungal community composition were identified by Voříšková et al. (2014). These changes are associated with nutrient input from fresh litter, which occurs in temperate deciduous forests each autumn (Voříšková et al., 2014). In accordance with our study, Lin et al. (2016) reported seasonal shifts of fungi in coniferous forests. The air and soil temperatures at both forest stands were higher in early summer, whereas the soil water content was increased in autumn (Table S6). Hence, comparable weather conditions would suggest similar fungal reactions toward changing season at the beech and spruce stand. However, a relatively thick needle litter layer (~8 cm) was removed before soil sampling under spruce. Breakdown of needles, which are highly recalcitrant to biological degradation, is mainly performed by fungi. It is possible that the distinct fungi colonizing needles (Korkama-Rajala et al., 2008) and consequently soil fungal communities under coniferous trees are susceptible to climatic changes in autumn. In addition, unmeasured factors might account for the shifts of fungal communities under spruce. Future studies can evaluate if these findings are artificial or ecologically reasonable.

## Conclusion

In accordance with our first hypothesis, beech and spruce trees strongly shaped the community composition of soil bacteria and fungi in temperate forests. Tree species-specific preferences with respect to bacterial and fungal microorganisms, such as a Chloroflexi representative, members of Acidobacteria subgroup 2 or *Hygrophorus* and *Clavulina*, were identified. Trees also have manifold impacts on the seasonal and spatial distribution of soil microorganisms. Indicator species analyses showed a vertical variation with a higher importance of saprotrophic taxa in the upper soil layer (0–10 cm) compared to the soil at a depth of 10–20 cm, supporting our second hypothesis. In line with our third hypothesis, bacterial community composition was strongly affected by tree distance, which might be due to higher fine root biomass near spruce trunks. Furthermore, bacterial community composition showed stronger seasonal variation under deciduous trees versus evergreen trees. This pattern was not found when analyzing fungal community composition, which is in contrast to our forth hypothesis. Noteworthy, soil fungal communities under spruce seem to be susceptible to seasonal changes. Overall, our results indicate that trees influence the spatial variation of bacteria and fungi, but their diverse patterns in stem flow, measured by pH change, seem to have a minor impact. Furthermore, the study indicates that soil pH and tree species (European beech or Norway spruce) have a stronger impact on soil bacterial and fungal community composition than soil depth, season or distance from tree trunk.

Additional studies considering root architecture and exudation patterns as well as the influence of tree canopy on the spatial distribution of leaf litter fall are necessary to further elucidate interactions between trees and soil microbes. Besides studies allowing analysis of the proportional importance of factors such as tree species, tree distance, or season, and their mechanisms for interaction, experimental designs focusing on effects of single factors are required to gain more comprehensive understanding on microbial community variation in forest soil. Furthermore, more direct proof is needed to ascertain functional roles of microbes such as Acidobacteria in soil surrounding beech and spruce. For instance, stable isotope probing could be used to identify bacteria or fungi involved in litter degradation.

## Author contributions

MS, FB, RD, and TW designed the study; HN, KG, IS, BP, KK, and GC carried out field and laboratory work; HN, KG, IS, and KK prepared and analyzed the data; all authors interpreted the results and wrote the paper.

### Conflict of interest statement

The authors declare that the research was conducted in the absence of any commercial or financial relationships that could be construed as a potential conflict of interest.

## Supplementary material

Table S1 **Information on selected trees including tree position, tree age, tree trunk circumference, and sampling direction away from the tree trunk in May and November 2012**.

Table S2 **Multivariate analysis of variance based on weighted UniFrac distances of whole bacterial and fungal community composition**.

Table S3 **Multivariate analysis of variance based on weighted UniFrac distances of bacterial and fungal community composition under beech or spruce**.

Table S4 **Analysis of covariance to test the impact of tree replicate, depth, season and distance from the tree trunk on soil chemical parameters under trees**.

Table S5 **Results of indicator species analysis showing potential indicative OTUs for tree species, sampling depths, sampling distances and sampling season under trees**.

Table S6 **Air temperature, soil temperature, and soil water content data**.

Figure S1 **Rarefaction curves indicating the observed numbers of OTUs at a genetic distance of 3%**. Samples derived from soil surrounding beech and samples collected under spruce are represented by brown and green color, respectively.

Figure S2 **Line plots showing soil bacterial and fungal diversity as assessed by Shannon index at 3% genetic distance under beech in (A)** early summer and **(C)** autumn, and under spruce in **(B)** early summer and **(D)** autumn.
