# Supplementary material for: Fine Spatial Scale Variation of Soil Microbial Communities under European Beech and Norway Spruce
Source: Front Microbiol. 2016 Dec 22;7:2067. doi: 10.3389/fmicb.2016.02067 (PMC5177625; doi:10.3389/fmicb.2016.02067)
Supplement: Table S1 — Information on selected trees including tree position, tree age, tree trunk circumference, and sampling direction away from the tree trunk in May and November 2012. [file Table1.DOCX]

**Table S1.** Information on selected trees including tree position, tree age, tree trunk circumference, and sampling direction away from the tree trunk in May and November 2012.

| Tree species | Replicate | Easting | Northing | Direction | Direction | Tree age | Trunk circumference |
| --- | --- | --- | --- | --- | --- | --- | --- |
|  |  | [m] | [m] | 1st sampling | 2nd sampling | [a] | (h=1.50m) |
|  |  |  |  | Early summer | Autumn |  | [m] |
|  |  |  |  | [°] | [°] |  |  |
| *Fagus sylvatica* | 1 | 3586543±5 | 5682178±5 | 135 | 118 | 55-65 | 1.56 |
| *Fagus sylvatica* | 2 | 3586530±5 | 5682187±5 | 182 | 165 | 55-65 | 1.2 |
| *Fagus sylvatica* | 3 | 3585534±5 | 5682178±5 | 293 | 285 | 55-65 | 1.34 |
| *Fagus sylvatica* | 4 | 3586530±5 | 5682162±5 | 35 | 20 | 55-65 | 1.4 |
| *Picea abies* | 1 | 3591581±7 | 5682660±7 | 195 | 213 | 50-60 | 1.38 |
| *Picea abies* | 2 | 3591567±7 | 5682668±7 | 350 | 5 | 50-60 | 1.09 |
| *Picea abies* | 3 | 3591595±7 | 5682693±7 | 5 | 0 | 50-60 | 1.22 |
| *Picea abies* | 4 | 3591594±7 | 5682652±7 | 210 | 195 | 50-60 | 1.26 |
