# Supplementary material for: Fine Spatial Scale Variation of Soil Microbial Communities under European Beech and Norway Spruce
Source: Front Microbiol. 2016 Dec 22;7:2067. doi: 10.3389/fmicb.2016.02067 (PMC5177625; doi:10.3389/fmicb.2016.02067)
Supplement: Table S2 — Multivariate analysis of variance based on weighted UniFrac distances of whole bacterial and fungal community composition. [file Table2.DOC]

Table S2. Multivariate analysis of variance based on weighted UniFrac distances of whole bacterial and fungal community composition. (a) Stepwise model testing for multivariate analysis of variance based on weighted UniFrac distances of whole bacterial and fungal community composition (128 samples) with seven considered response factors in the order of entering the analysis. Increasing *R²*-values, and adjusted *R²*-values represent chosen model suitability. (b) Analysis of final model with seven response variables. *R²* and adjusted *R²* for each variable within the model. Explanatory variables are given in rows in the order of entering the analysis. Significant results are indicated by **P* < 0.05, ***P* < 0.01, ****P* < 0.001. Abbreviation: Adj., Adjusted.

(a)

| No. of factors | Factor | Bacterial community | | Fungal community | |
| --- | --- | --- | --- | --- | --- |
|  |  | *R²* | Adj. *R²* | *R²* | Adj. *R²* |
| 1 | Replicate | 0.064 | 0.056 | 0.107 | 0.100 |
| 2 | pH | 0.206 | 0.193 | 0.166 | 0.153 |
| 3 | OC | 0.247 | 0.229 | 0.185 | 0.165 |
| 4 | Depth | 0.265 | 0.241 | 0.200 | 0.174 |
| 5 | Season | 0.282 | 0.253 | 0.225 | 0.193 |
| 6 | Distance | 0.311 | 0.276 | 0.239 | 0.201 |
| 7 | Tree species | 0.449 | 0.417 | 0.388 | 0.352 |

(b)

|  |  | Bacterial community | | | Fungal community | | |
| --- | --- | --- | --- | --- | --- | --- | --- |
|  | *df* | *MS* | *R²* | Adj. *R²* | *MS* | *R²* | Adj. *R²* |
| Replicate | 3 | 0.126 | 0.064*** | 0.009 | 1.808 | 0.107*** | 0.055 |
| pH | 1 | 0.844 | 0.142*** | 0.092 | 3.016 | 0.059*** | 0.004 |
| OC | 1 | 0.245 | 0.041*** | 0.000 | 0.940 | 0.019*** | 0.000 |
| Depth | 1 | 0.103 | 0.017** | 0.000 | 0.740 | 0.015** | 0.000 |
| Season | 1 | 0.103 | 0.017** | 0.000 | 1.271 | 0.025*** | 0.000 |
| Distance | 1 | 0.170 | 0.029*** | 0.000 | 0.706 | 0.014** | 0.000 |
| Tree species | 1 | 0.821 | 0.138*** | 0.088 | 7.555 | 0.149*** | 0.099 |
