# Supplementary material for: Fine Spatial Scale Variation of Soil Microbial Communities under European Beech and Norway Spruce
Source: Front Microbiol. 2016 Dec 22;7:2067. doi: 10.3389/fmicb.2016.02067 (PMC5177625; doi:10.3389/fmicb.2016.02067)
Supplement: Table S3 — Multivariate analysis of variance based on weighted UniFrac distances of bacterial and fungal community composition under beech or spruce. [file Table3.DOC]

Table S3. Multivariate analysis of variance based on weighted UniFrac distances of bacterial and fungal community composition under beech or spruce. (a) Stepwise model testing for multivariate analysis of variance based on weighted UniFrac distances of bacterial and fungal community composition under beech or spruce (64 samples per tree species) with six considered response factors in the order of entering the analysis. Increasing *R²*-values, and adjusted *R²*-values represent chosen model suitability. (b) Analysis of final model with six response variables. *R²* and adjusted *R²* for each variable within the model. Explanatory variables are given in rows in the order of entering the analysis (b). Significant results are indicated by **P* < 0.05, ***P* < 0.01, ****P* < 0.001. Abbreviation: Adj., Adjusted.

(a)

| No. of factors | Factor | Beech stand | | | |  |  | Spruce stand | |  |
| --- | --- | --- | --- | --- | --- | --- | --- | --- | --- | --- |
|  |  | Bacterial community | | Fungal community | |  | Bacterial community | | Fungal community | |
|  |  | *R²* | Adj. *R²* | *R²* | Adj. *R²* |  | *R²* | Adj. *R²* | *R²* | Adj. *R²* |
| 1 | Replicate | 0.063 | 0.047 | 0.233 | 0.221 |  | 0.171 | 0.158 | 0.323 | 0.312 |
| 2 | pH | 0.234 | 0.209 | 0.265 | 0.241 |  | 0.281 | 0.257 | 0.370 | 0.350 |
| 3 | OC | 0.330 | 0.297 | 0.305 | 0.271 |  | 0.300 | 0.265 | 0.394 | 0.363 |
| 4 | Depth | 0.343 | 0.298 | 0.319 | 0.272 |  | 0.329 | 0.283 | 0.406 | 0.366 |
| 5 | Season | 0.403 | 0.352 | 0.340 | 0.283 |  | 0.351 | 0.295 | 0.426 | 0.377 |
| 6 | Distance | 0.424 | 0.363 | 0.360 | 0.293 |  | 0.425 | 0.364 | 0.459 | 0.402 |

(b)

|  | Beech stand | | | |  | Spruce stand | | | |
| --- | --- | --- | --- | --- | --- | --- | --- | --- | --- |
|  | Bacterial community | | Fungal community | |  | Bacterial community | | Fungal community | |
|  | *R²* | Adj*. R²* | *R²* | Adj. *R²* |  | *R²* | Adj. *R²* | *R²* | Adj. *R²* |
| Tree replicate | 0.063** | 0.000 | 0.233*** | 0.152 |  | 0.171*** | 0.084 | 0.323*** | 0.252 |
| pH | 0.171*** | 0.084 | 0.032** | 0.000 |  | 0.109*** | 0.015 | 0.048*** | 0.000 |
| OC | 0.097*** | 0.002 | 0.04*** | 0.000 |  | 0.020 | 0.000 | 0.023** | 0.000 |
| Depth | 0.013 | 0.000 | 0.013 | 0.000 |  | 0.028* | 0.000 | 0.013 | 0.000 |
| Season | 0.06*** | 0.000 | 0.021 | 0.000 |  | 0.022 | 0.000 | 0.02* | 0.000 |
| Distance | 0.021* | 0.000 | 0.020 | 0.000 |  | 0.074*** | 0.000 | 0.032*** | 0.000 |
