# Supplementary material for: Fine Spatial Scale Variation of Soil Microbial Communities under European Beech and Norway Spruce
Source: Front Microbiol. 2016 Dec 22;7:2067. doi: 10.3389/fmicb.2016.02067 (PMC5177625; doi:10.3389/fmicb.2016.02067)
Supplement: Table S4 — Analysis of covariance to test the impact of tree replicate, depth, season and distance from the tree trunk on soil chemical parameters under trees. [file Table4.DOCX]

**Table S4.** Analysis of covariance to test the impact of tree replicate, depth, season and distance from the tree trunk on soil chemical parameters under trees. Samples collected under beech (a) and spruce (b) were analyzed. Significant results are indicated by **P* < 0.05, ***P* < 0.01, ****P* < 0.001. In this table degrees of freedom (*df*), mean squares (*MS*), and *F*-values are presented.

(a)

|  | *df* | pH |  | OC |  | N |  | C:N |  | Clay |  |
| --- | --- | --- | --- | --- | --- | --- | --- | --- | --- | --- | --- |
|  |  | *MS* | *F* | *MS* | *F* | *MS* | *F* | *MS* | *F* | *MS* | *F* |
| Tree replicate | 3 | 0.03 | 2.8* | 0.01 | 0.1 | 0.00 | 0.1 | 0.12 | 0.4 | 3351 | 13.7*** |
| Depth | 1 | 0.68 | 55.7*** | 22.15 | 230.7*** | 0.12 | 233.2*** | 17.67 | 63.2*** | 12324 | 50.5*** |
| Season | 1 | 0.01 | 0.5 | 0.46 | 4.8* | 0.00 | 2.9 | 1.35 | 4.8* | 2680 | 11.0** |
| Distance | 1 | 0.02 | 1.2 | 3.11 | 32.4*** | 0.02 | 30.8*** | 3.48 | 12.5*** | 10226 | 41.9*** |
| Residuals | 57 | 0.01 |  | 0.10 |  | 0.00 |  | 0.28 |  | 244 |  |

(b)

|  | *df* | pH |  | OC |  | N |  | C:N |  | Clay |  |
| --- | --- | --- | --- | --- | --- | --- | --- | --- | --- | --- | --- |
|  |  | *MS* | *F* | *MS* | *F* | *MS* | *F* | *MS* | *F* | *MS* | *F* |
| Tree replicate | 3 | 18.96 | 93.0*** | 5.02 | 7.4*** | 0.02 | 10.7*** | 3.67 | 2.9* | 84533 | 30.2*** |
| Depth | 1 | 12.87 | 63.1*** | 49.23 | 72.6*** | 0.11 | 56.3*** | 226.48 | 181.1*** | 1133 | 0.4 |
| Season | 1 | 1.65 | 8.1** | 0.53 | 0.8 | 0.00 | 0.1 | 15.47 | 12.4*** | 308 | 0.1 |
| Distance | 1 | 1.61 | 7.9** | 2.59 | 3.8 | 0.00 | 0.6 | 16.63 | 13.3*** | 11888 | 4.2* |
| Residuals | 57 | 0.20 |  | 0.68 |  | 0.00 |  | 1.25 |  | 2800 |  |
