# Supplementary material for: Fine Spatial Scale Variation of Soil Microbial Communities under European Beech and Norway Spruce
Source: Front Microbiol. 2016 Dec 22;7:2067. doi: 10.3389/fmicb.2016.02067 (PMC5177625; doi:10.3389/fmicb.2016.02067)
Supplement: Table S5 — Results of indicator species analysis showing potential indicative OTUs for tree species, sampling depths, sampling distances and sampling season under trees. [file Table5.DOC]

Table S5. Results of indicator species analysis showing potential indicative OTUs for tree species, sampling depths, sampling distances and sampling season under trees. Bacterial communities under beech (a) and spruce (b) as well as fungal communities under beech (c) and spruce (d) were analyzed. Statistical values: A - species only occurs in sites of this group, B - all sites of this group harbor this species, cutoff (A+B) = 0.7, *P* - significance value. Relative abundances of OTUs across all 128 samples are depicted. Abbreviations: Rel. abundance, Relative abundance; Acidob., Acidobacteria.

(a)

| Indicator group | Bacterial OTU | A | B | *P* | Rel. abundance (%) |
| --- | --- | --- | --- | --- | --- |
| Tree species | | | | | |
| Beech | Xanthomonadales | 0.99431 | 1 | 0.005 | 0.216 |
|  | Rhodospirillales | 0.99403 | 1 | 0.005 | 0.257 |
|  | Acidob. subgroup 1 | 0.99449 | 0.98438 | 0.005 | 0.112 |
|  | Acidob. subgroup 2 | 0.98855 | 0.98438 | 0.005 | 0.081 |
|  | Xanthomonadales | 0.9919 | 0.96875 | 0.005 | 0.076 |
|  | Acidob. subgroup 3 (*Bryobacter*) | 0.95113 | 1 | 0.005 | 0.422 |
|  | Gemmatimonadaceae | 0.96051 | 0.98438 | 0.005 | 0.241 |
|  | Acidob. subgroup 2 | 0.97674 | 0.95312 | 0.005 | 0.132 |
|  | Acidob. subgroup 3 (*Bryobacter*) | 0.93072 | 1 | 0.005 | 0.480 |
|  | Acidob. subgroup 2 | 0.98697 | 0.9375 | 0.005 | 0.094 |
|  | Acidob. subgroup 2 | 0.98664 | 0.9375 | 0.005 | 0.322 |
|  | Acidob. subgroup 2 | 0.90485 | 1 | 0.005 | 0.818 |
|  | *Gemmatimonas* | 0.93322 | 0.96875 | 0.005 | 0.184 |

Table S5a (continued)

| Indicator group | Bacterial OTU | A | B | *P* | Rel. abundance (%) |
| --- | --- | --- | --- | --- | --- |
| Depths | | | | | |
| 10-20 cm | Chloroflexi | 0.8966 | 0.8125 | 0.005 | 0.027 |
|  | Acidob. subgroup 1 | 0.8226 | 0.7812 | 0.005 | 0.019 |
|  | Nitrospirales | 0.7877 | 0.8125 | 0.005 | 0.045 |
| Distances | | | | |  |
| 0.5 + 1.5 + 2.5 m | *Gemmatimonas* | 0.8125 | 0.7083 | 0.005 | 0.044 |
| 1.5 + 2.5 + 3.5 m | *Bryobacter* | 0.8841 | 0.8542 | 0.005 | 0.042 |
|  | Betaproteobacterium | 0.86 | 0.875 | 0.005 | 0.062 |
| Season | | | | |  |
| Early summer | *Bryobacter* | 0.8686 | 0.75 | 0.005 | 0.042 |
|  | *Bryobacter* | 0.8323 | 0.7188 | 0.005 | 0.048 |
| Autumn | *Mycobacterium* | 0.8065 | 0.9375 | 0.005 | 0.048 |
|  | *Bradyrhizobium* | 1 | 0.75 | 0.005 | 0.038 |
|  | Xanthobacteraceae | 0.9478 | 0.75 | 0.005 | 0.035 |

(b)

| Indicator group | Bacterial OTU | A | B | *P* | Rel. abundance (%) |
| --- | --- | --- | --- | --- | --- |
| Tree species | | | | | |
| Spruce | Rhodospirillales | 0.9918 | 0.85938 | 0.005 | 0.188 |
|  | Acidob. subgroup 1 | 0.9519 | 0.82812 | 0.005 | 0.332 |
|  | Xanthomonadales | 0.98683 | 0.79688 | 0.005 | 0.537 |
|  | Rhodospirillales | 0.93238 | 0.78125 | 0.005 | 0.086 |
|  | Acidob. subgroup 1 | 0.9065 | 0.79688 | 0.005 | 0.151 |
|  | Acidob. subgroup 1 | 0.88484 | 0.8125 | 0.005 | 0.160 |
|  | Acidob. subgroup 1 | 0.95414 | 0.75 | 0.005 | 0.174 |
|  | Chloroflexi | 0.97448 | 0.71875 | 0.005 | 0.313 |
|  | WD272 | 0.94089 | 0.73438 | 0.005 | 0.062 |
|  | Acidob. subgroup 3 (*Bryobacter*) | 0.94694 | 0.71875 | 0.005 | 0.075 |
| Depths | | | | |  |
| 0-10 cm | Xanthomonadales | 0.8868 | 0.7812 | 0.005 | 0.114 |
|  | Acetobacteraceae | 0.7201 | 0.8438 | 0.005 | 0.082 |
|  | Xanthomonadales | 0.8293 | 0.7188 | 0.005 | 0.025 |
|  | *Derxia* | 0.8219 | 0.7188 | 0.005 | 0.067 |

Table S5b (continued)

| Indicator group | Bacterial OTU | A | B | *P* | Rel. abundance (%) |
| --- | --- | --- | --- | --- | --- |
| Depths | | | | |  |
| 10-20 cm | Gaiellales | 0.7901 | 0.8438 | 0.005 | 0.100 |
|  | Gaiellales | 0.8485 | 0.7812 | 0.005 | 0.112 |
|  | *Caldilinea* | 0.7536 | 0.8125 | 0.005 | 0.086 |
|  | Acidimicrobiales | 0.715 | 0.7812 | 0.005 | 0.064 |
| Distances | | | | |  |
| 2.5 + 3.5 m | Nitrospirales | 0.8109 | 0.7188 | 0.005 | 0.192 |
| 0.5 + 1.5 + 2.5 m | WD272 | 0.9549 | 0.7708 | 0.005 | 0.198 |

(c)

| Indicator group | Fungal OTU | A | B | *P* | Rel. abundance (%) |
| --- | --- | --- | --- | --- | --- |
| Tree species | | | | | |
| Beech | *Mortierella elongata* | 0.9987 | 1 | 0.005 | 0.598 |
|  | Mortierellomycetes | 0.9968 | 0.9688 | 0.005 | 0.253 |
|  | Coniochaetaceae | 0.9657 | 0.9219 | 0.005 | 0.175 |
|  | fungal OTU | 0.8301 | 0.875 | 0.005 | 0.160 |
|  | *Trichoderma* | 0.9271 | 0.8594 | 0.005 | 0.125 |
|  | *Xerocomus chrysenteron* | 1 | 0.8125 | 0.005 | 0.324 |
|  | *Russula cyanoxantha* | 0.9987 | 0.8125 | 0.005 | 9.558 |
|  | Helotiales | 0.9818 | 0.7656 | 0.005 | 0.065 |
| Depths | | | | |  |
| 0-10 cm | *Varicosporium* | 0.8513 | 0.7813 | 0.005 | 0.053 |
|  | *Leptodontidium* | 0.6958 | 0.75 | 0.005 | 0.045 |
|  | *Volutella* | 0.8587 | 0.6875 | 0.005 | 0.036 |
|  | Didymellaceae | 0.8181 | 0.6875 | 0.005 | 0.026 |
|  | fungal OTU | 0.7895 | 0.6875 | 0.005 | 0.028 |
| Distance | | | | |  |
| 0.5 m + 1.5 m | *Leptodontidium elatius* | 0.7635 | 0.6562 | 0.005 | 0.025 |
| 2.5 m + 3.5 m | *Russula cyanoxantha* | 0.9749 | 0.5312 | 0.005 | 2.140 |
| 0.5 m + 1.5 m + 2.5 | *Piloderma* | 0.9398 | 0.5208 | 0.005 | 0.091 |
| 1.5 m + 2.5 m + 3.5 m | *Leotia* | 0.9179 | 0.6667 | 0.005 | 0.641 |
|  | *Inocybe* | 0.9474 | 0.5208 | 0.005 | 1.504 |

Table S5c (continued)

| Indicator group | Fungal OTU | A | B | *P* | Rel. abundance (%) |
| --- | --- | --- | --- | --- | --- |
| Distance | | | | | |
| 0.5 m + 1.5 m + 2.5 m + 3.5 m | *Mortierella elongata* | 0.9987 | 1 | 0.005 | 0.598 |
|  | Mortierellomycetes | 0.9968 | 0.9688 | 0.005 | 0.253 |
|  | Coniochaetaceae | 0.9657 | 0.9219 | 0.005 | 0.175 |
|  | *Xerocomus chrysenteron* | 0.8301 | 0.875 | 0.005 | 0.324 |
|  | *Russula cyanoxantha* | 0.9271 | 0.8594 | 0.005 | 9.558 |
|  | *Trichoderma* | 1 | 0.8125 | 0.005 | 0.125 |
|  | Helotiales | 0.9987 | 0.8125 | 0.005 | 0.065 |
|  | fungal OTU | 0.9818 | 0.7656 | 0.005 | 0.160 |
| Season | | | | |  |
| Early summer | *Russula cyanoxantha* | 0.9979 | 0.5 | 0.005 | 0.186 |
| Autumn | Helotiales | 0.9936 | 1 | 0.005 | 0.368 |
|  | Mortierellaceae | 0.7546 | 0.9688 | 0.005 | 0.094 |
|  | *Mycospaerella* | 1 | 0.7188 | 0.005 | 0.027 |

(d)

| Indicator group | Fungal OTU | A | B | *P* | Rel. abundance (%) |
| --- | --- | --- | --- | --- | --- |
| Tree species | | | | | |
| Spruce | *Exophiala* | 0.989 | 0.9688 | 0.005 | 0.178 |
|  | *Penicillium inflatu*m | 0.9091 | 0.9688 | 0.005 | 0.095 |
|  | *Hygrophorus discoideus* | 0.9995 | 0.7969 | 0.005 | 3.169 |
|  | Mortierellales | 1 | 0.7813 | 0.005 | 0.087 |
|  | *Hygrophorus pustulatus* | 0.9997 | 0.7656 | 0.005 | 2.338 |
|  | Helotiales | 0.9981 | 0.7656 | 0.005 | 0.203 |
|  | *Penicillium* | 1 | 0.75 | 0.005 | 0.131 |
|  | *Amphinema* | 0.9986 | 0.7188 | 0.005 | 1.387 |
| Depths | | | | |  |
| 0-10 cm | Helotiales | 0.8581 | 0.9375 | 0.005 | 0.203 |
|  | *Mortierella* | 0.7384 | 0.7813 | 0.005 | 0.066 |
| 10-20 cm | *Cryptococcus fuscescens* | 0.7656 | 0.7813 | 0.005 | 0.102 |
|  | *Mortierella* | 0.8424 | 0.75 | 0.005 | 0.068 |
|  | *Leohumicola* | 0.9557 | 0.5313 | 0.005 | 0.018 |
| Distance | | | | |  |
| 0.5 m + 1.5 m + 2.5 | *Russula* | 0.9866 | 0.7292 | 0.005 | 4.941 |
| 0.5 m + 1.5 m + 2.5 m + 3.5 m | *Exophiala* | 0.989 | 0.9688 | 0.005 | 0.178 |
|  | *Penicillium inflatum* | 0.9091 | 0.8125 | 0.005 | 0.095 |
|  | *Hygrophorus discoideus* | 0.9995 | 0.7969 | 0.005 | 3.169 |
|  | Mortierellales | 1 | 0.7812 | 0.005 | 0.087 |
|  | Helotiales | 0.7812 | 0.7812 | 0.005 | 0.203 |
|  | *Penicillium* | 1 | 0.75 | 0.005 | 0.131 |
|  | *Amphinema* | 0.9986 | 0.7188 | 0.005 | 1.387 |
| Season | | | | |  |
| Early summer | Mortierellales | 0.9837 | 0.625 | 0.005 | 0.024 |
|  | *Amphinema* | 1 | 0.5625 | 0.005 | 0.168 |
