# Supplementary material for: Fine Spatial Scale Variation of Soil Microbial Communities under European Beech and Norway Spruce
Source: Front Microbiol. 2016 Dec 22;7:2067. doi: 10.3389/fmicb.2016.02067 (PMC5177625; doi:10.3389/fmicb.2016.02067)
Supplement: Table S6 — Air temperature, soil temperature, and soil water content data. [file Table6.DOC]

Table S6. Air temperature, soil temperature, and soil water content data. Five year averages (2008-2012) are presented.

| Time | Air temperature (2 m height) | |  | Soil temperature (10 cm depth) | |  | Volumetric water content (10 cm depth) | |
| --- | --- | --- | --- | --- | --- | --- | --- | --- |
|  | Beech forest | Spruce forest |  | Beech forest | Spruce forest |  | Beech forest | Spruce forest |
|  | (°C) | |  | (°C) | |  | (%) | |
| May | 12.1 | 12.7 |  | 10.1 | 10.6 |  | 28.0 | 23.5 |
| November | 4.1 | 4.2 |  | 6.5 | 7.0 |  | 31.5 | 25.2 |
| January-December | 7.3 | 7.7 |  | 8.0 | 8.4 |  | 29.3 | 25.4 |
