# Supplementary figures and images for: Fine Spatial Scale Variation of Soil Microbial Communities under European Beech and Norway Spruce
Source: Front Microbiol. 2016 Dec 22;7:2067. doi: 10.3389/fmicb.2016.02067 (PMC5177625; doi:10.3389/fmicb.2016.02067)

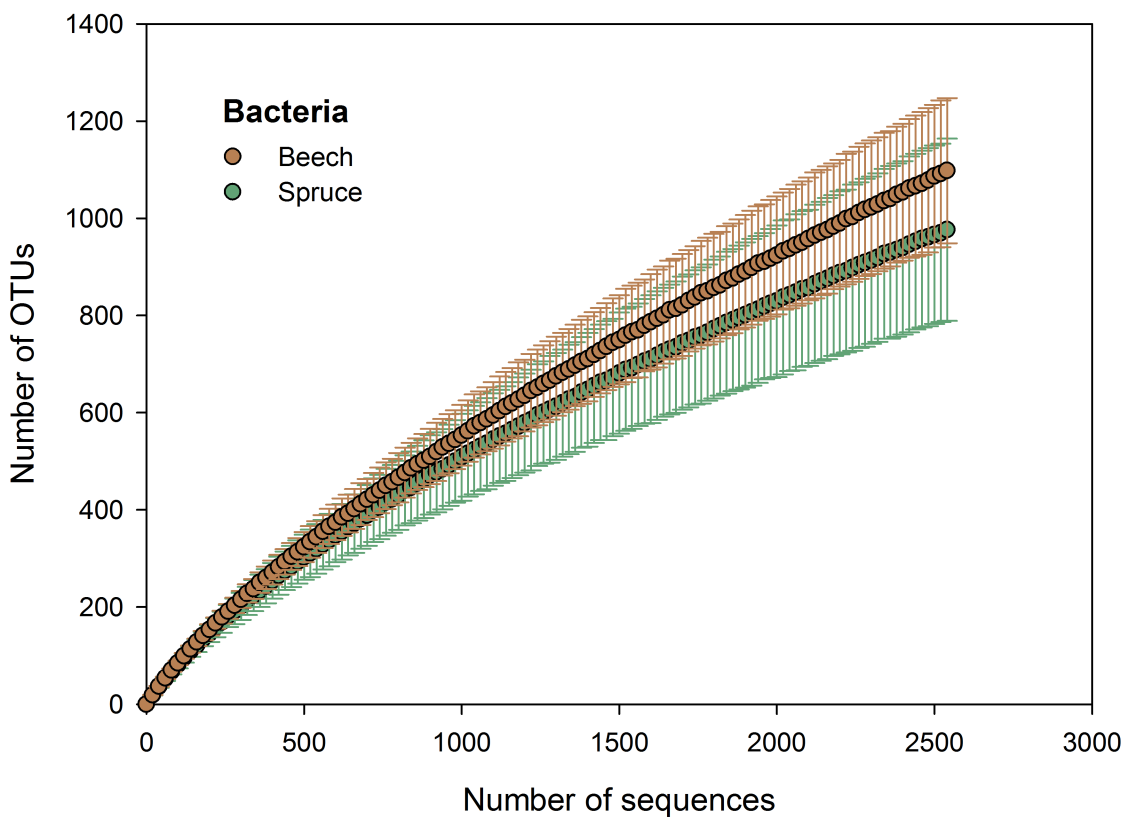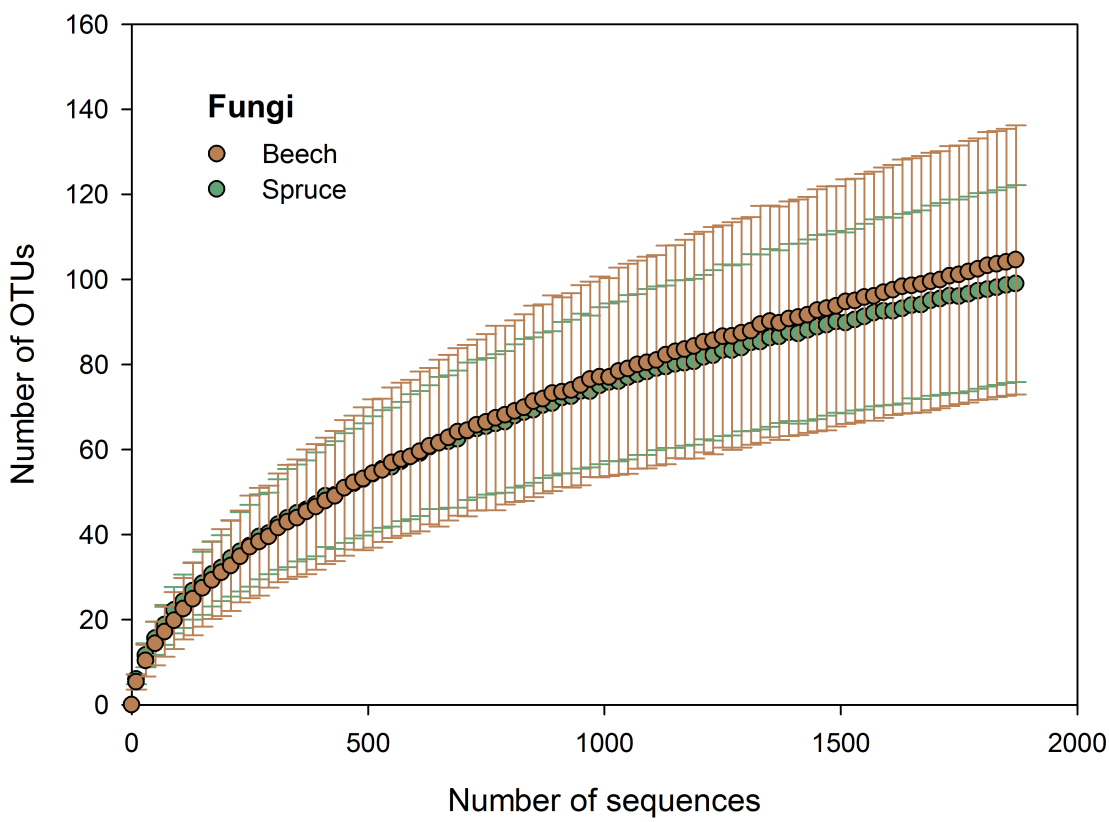

Supplement: Figure S1 — Rarefaction curves indicating the observed numbers of OTUs at a genetic distance of 3%. Samples derived from soil surrounding beech and samples collected under spruce are represented by brown and green color, respectively. [file Image1.PDF]

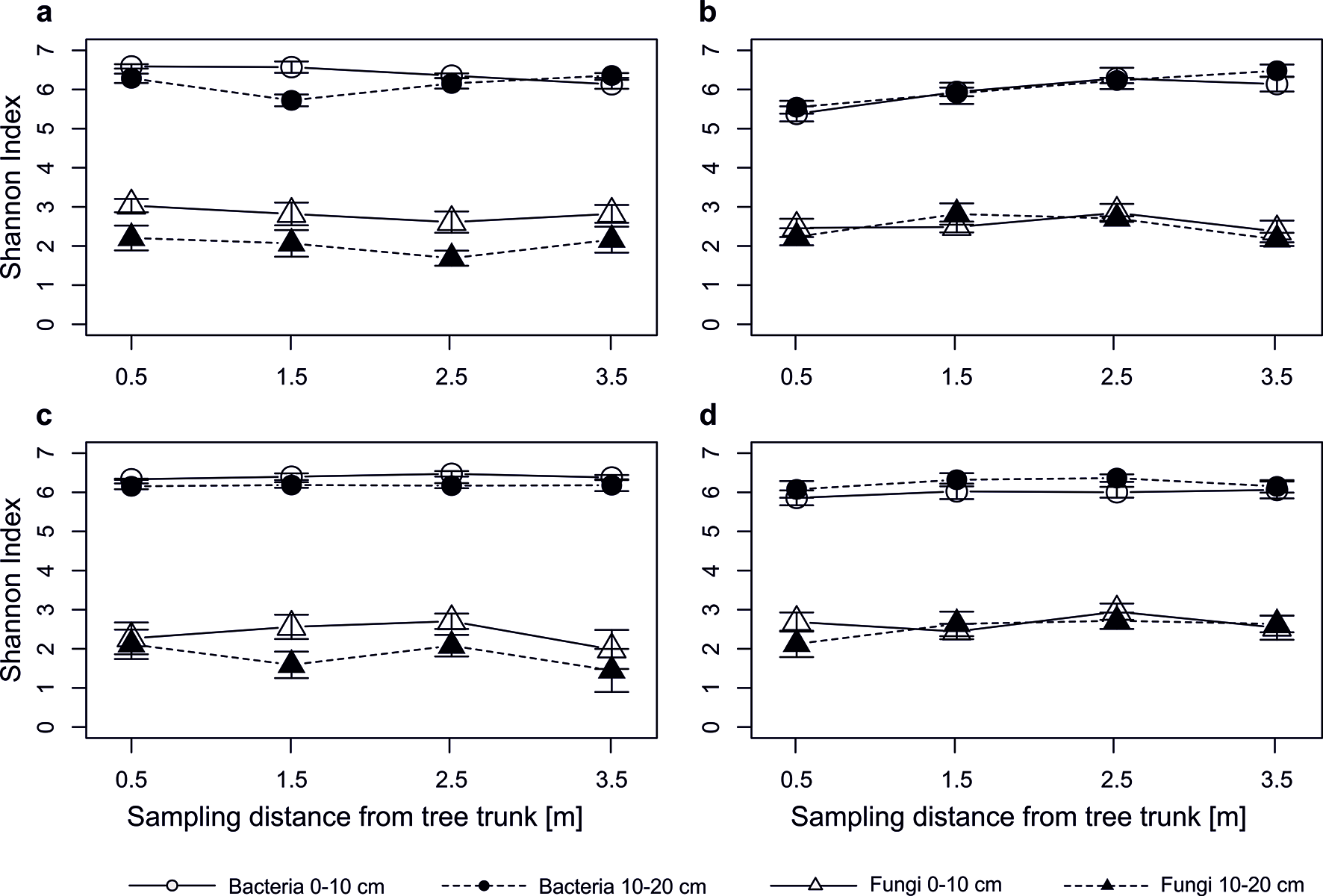

Supplement: Figure S2 — Line plots showing soil bacterial and fungal diversity as assessed by Shannon index at 3% genetic distance under beech in (A) early summer and (C) autumn, and under spruce in (B) early summer and (D) autumn. [file Image2.TIF]
